# Ethanol Extract of* Lycopus lucidus* Turcz. ex Benth Inhibits Metastasis by Downregulation of Runx-2 in Mouse Colon Cancer Cells

**DOI:** 10.1155/2018/9513290

**Published:** 2018-07-19

**Authors:** Kwang-Youn Kim, Tae Woo Oh, Jin-Yeul Ma, Kwang-Il Park

**Affiliations:** Korean Medicine (KM) Application Center, Korea Institute of Oriental Medicine (KIOM), Daegu 41062, Republic of Korea

## Abstract

*Lycopus lucidus *Turcz. ex Benth (LT) has been broadly used as a traditional medicinal herb in Asia including Korea, China, and Japan due to its noted ability to promote blood circulation and remove blood stasis. However, its anticancer mechanism is not understood. This study aims to elucidate the effects of ethanol extracts of LT (ELT) relative to the role of Runt-related transcription factor- (Runx-) 2 in the invasive and metastatic potentials of mouse colon cancer to determine the underlying mechanisms involved. ELT was evaluated for the antimetastasis activity using CT-26 colon cancer using wound healing, transwell matrigel, and western blot analysis. We used Runx-2-specific siRNA to further determine the relationship between Runx-2 and matrix metalloprotease- (MMP-) 9 in the migration and invasion of CT-26 cells. Runx-2 was first demonstrated to be a transcription factor that plays a remarkable role in diverse biological processes of chondrocytes and osteoblasts, but recently, Runx-2 has been reported to be associated with the progression of certain human cancers. ELT was not altered in its effects on growth inhibition. However, ELT significantly inhibited wound closure and cell invasion in a dose-dependent manner. ELT decreased the metastasis by regulating the activity of MMP-9 and Runx-2 at the translational levels. Our results demonstrate that ELT decreases metastasis by inhibiting the Runx-2–MMP-9 axis. We suggest that it can be used as a novel agent in therapeutic strategies for combating colon cancer.

## 1. Introduction

Colon cancer is a malignant type of cancer that affects both men and women and is the third leading cause of death in the United States [1]. Recently, surgical resection and chemotherapy have been used to prolong the survival of patients with cancer [2]; however, such results remain unsatisfactory, and the recurrence rate after surgery remains high [3, 4]. Therefore, further studies are required to investigate the processing mechanism(s) underlying the disease to improve clinical outcomes.

The Runt-related transcription factor-2 (Runx-2) was initially reported to be an important factor in the diverse processes of differentiation and migration of chondrocyte and osteoblast cells [5, 6]. Runx-2 is being increasingly recognized as regulating the development of cancer and its progression [7]. Several reports have shown that the high expressions of Runx-2 are obviously correlated with high histological grade, multiple tumor nodes, and tumor infiltration and metastasis [7, 8]. Furthermore, Runx-2 has been found to promote osteolytic and skeletal destruction by enhancing metastasis-related protein expressions, such as matrix metalloproteinase- (MMP-) 9 and MMP-13 found in prostate and breast cancers [9–11].

*Lycopus lucidus *Turcz. ex Benth (LT) is a perennial herb widely used as a traditional medicine in Korea and China to promote blood circulation and remove blood stasis [12–14]. Recently, it has been reported that LT both inhibits vascular inflammation in human umbilical vein endothelial cells and reduces mast cell-mediated allergies [15, 16]. LT can improve renal intestinal fibrosis and decrease heart rates in rats. Furthermore, it has been shown to improve the immune system in mice [16, 17]. Also, LT has anticancer and antioxidant activities in liver carcinoma and breast cancer cell lines as well as antimicrobial activity in microorganisms [18]. Although research in a diversity of fields has demonstrated the effects of LT, the underlying molecular mechanisms related to anticancer and antimetastasis remain unknown. Therefore, this study aimed to elucidate the effects of ethanol extracts of LT (ELT) relative to the role of Runx-2 in the metastatic and invasive potentials of mouse colon cancer CT-26 cells and to reveal the underlying mechanisms involved. Our findings will determine whether ELT can be used as a novel therapeutic agent in the treatment of colon cancer.

## 2. Material and Methods

### 2.1. Preparation of ELT

LT was obtained from Yeongcheon Oriental Herbal Market (Yeongcheon, Korea) and authenticated by Professor Ki Hwan Bae, a medical botanist at the College of Pharmacy, Chungnam National University, Republic of Korea. Dried LT (30 g) was ground to a fine powder, added to 390 ml of 70% ethanol, and then extracted by shaking it in an incubator for 24 h. The extract was then filtered through a testing sieve (150 *μ*m, Retsch, Haan, Germany), evaporated and concentrated through lyophilization, and then stored at -20°C (yield 4.62%). For experiments, ELT powder (10 mg) was dissolved in 1 ml of deionized distilled water (v/v) and filtered through a 0.22 *μ*m disk filter.

### 2.2. High-Performance Liquid Chromatography (HPLC) Analysis

ELT and standard samples (protocatechuic acid, caffeic acid, rutin, and rosmarinic acid) were verified using a Dionex HPLC System (Dionex Co., Sunnyvale, CA, USA), equipped with an ultimate 3000 series binary pump, an autosampler, a column oven, and a diode array UV/VIS detector. Data were analyzed with Dionex Chromeleon software. All chromatographic separations were performed through an OptimaPak C18 column (5 *μ*m, 4.6 × 250 mm; Phenomenex, Torrance, CA, USA), with the temperature of the column maintained at 40°C. The mobile phase comprised 0.1% formic acid (v/v) in water (A) and acetonitrile (B). The linear gradient elution system (at a flow rate of 1.0 ml/min) was programmed as follows: 5% (B) for 0–5 min, 5–35% (B) for 5–35 min, 35% (B) for 35–45 min, 35–70% (B) for 45–70 min, 70% (B) for 70–75 min, 70–75% (B) for 75–75.5 min, 5% (B) for 75.5–85 min. The detection wavelengths for the four components were set at 203, 254, 280, and 330 nm.

### 2.3. Cell Lines and Culture

A mouse colon cancer CT-26 cell line was obtained from ATCC (Manassas, VA, USA). Cells were cultured at 37°C in a humidified atmosphere with 5% CO_2_ using Dulbecco's modified Eagle's medium (DMEM; HyClone, Logan, UT, USA) containing 10% fetal bovine serum (FBS; Hyclone), 100 units/ml penicillin, and 100 *μ*g/ml streptomycin (HyClone)

### 2.4. Cell Viability

The cytotoxicity of ELT on CT-26 cells was calculated using a cell counting kit-8 (CCK-8) assay (Dojindo Molecular Technologies, Inc., Rockville, MD, USA). The cells were seeded at 1 × 10^4^ cells/well in a 96-well plate. After 24 h incubation, the cells in each well were treated with ELT at specific concentrations for 24 h. The CCK-8 assay was then performed in accordance with the manufacturer's instructions. Absorbance was determined at 450 nm on a VERSAmax microplate reader (Molecular Devices, Sunnyvale, CA, USA). Cell viability was calculated relative to untreated controls [i.e., viability (% control) = 100 × absorbance of treated sample/absorbance of control].

### 2.5. Wound Healing Assay

Cells were seeded at 1 × 10^4^ cells/well in a 6-well plate. After 24 h incubation, the cells were pretreated with 25 *μ*g/ml of mitomycin C (Sigma Aldrich, St. Louis, MO, USA) for 30 min, after which wound lines were drawn on a confluent monolayer of cells. After washing with DMEM, the cells were treated with ELT at the specified concentrations for 24 h, and migration was then observed under a phase-contrast microscope.

### 2.6. Transwell Invasion Assays

For the invasion assay, CT-26 cells were tested in a transwell polycarbonate-membrane chamber (10-mm diameter and an 8-*μ*m pore size) (Corning Costar, Cambridge, MA, USA) after coating with 20 *μ*L of a 1:2 mixture of Matrigel:DMEM (Matrigel; BD Biosciences, Bedford, MA, USA). This formed an intervening, invasive barrier. CT-26 cells suspended in serum-free DMEM were then loaded onto the top chamber (1×10^4^ cells/insert), and ELT was added at the specified concentrations. Complete DMEM with 10% FBS was used in the lower chamber as a chemoattractant. After 24 h incubation, cells attached in the upper surface of filters were removed by wiping the filters with a cotton swab, and the filters were then stained with a 0.2% crystal violet/20% methanol (wt/vol) solution.

### 2.7. Gelatin Zymography

After preincubating cells with serum-free DMEM for 18 h, cells were treated with ELT for 24 h. Equal volumes of the ELT treated medium and sodium dodecyl sulfate (SDS) sample buffer (without reducing agents) were mixed and separated with 10% SDS-polyacrylamide gel (PAGE) containing 0.1% gelatin. Gels were then washed with washing buffer (50 mM Tris-HCl, pH 7.5; 100 mM NaCl; and 2.5% Triton X-100), rinsed twice with water, and then incubated at 37°C for 36 h in developing buffer (50 mM Tris-HCl, pH 7.5; 150 mM NaCl; 10 mM CaCl_2_; 0.02% NaN_3_; and 1 *μ*M ZnCl_2_). The gels were stained with Coomassie Brilliant Blue R-250 staining solution (Bio-Rad Laboratories, Hercules, CA, USA) and then destained (with a mixture of 10% isopropanol and 10% acetic acid). MMP-9 was detected as clear bands against a dark blue background.

### 2.8. RNA Interference

Runx-2 specific siRNA (ON-TARGET plus mouse Runx-2) was purchased from Dharmacon (Lafayette, CO, USA). Cells were transfected with a fresh medium (without serum) of 10 nM siRNA and Lipofectamine RNAiMAX reagent (Invitrogen, Carlsbad, CA, USA) for 24 h (until analysis) according to the manufacturer's instructions.

### 2.9. Western Blot Analysis

Cells were extracted using a lysis buffer [150 mM NaCl, 10 mM Tris (pH 7.4), 5 mM EDTA (pH 8.0), 1% Triton X-100, 1 mM PMSF, 20 mg/ml aprotinin, 50 *μ*g/ml leupeptin, 1 mM benzidine, 1 mg/ml pepstatin, 8 mM sodium pyrophosphate, and 20 mM *β*-glycerophosphate]. Forty micrograms of proteins were electrophoretically separated with 8–15% SDS-PAGE gel and transferred to a nitrocellulose membrane. After blocking with TBS-T buffer [20 mM Tris (pH 7.4), 150 mM NaCl, and 0.1% Tween 20] containing 5% skim milk, membranes were incubated at 4°C for overnight with primary antibodies (Runx-2, MMP-9, and *β*-actin; Cell Signaling Technology, Danvers, MA, USA). Membranes were washed with TBS-T buffer and then incubated at room temperature for 2 h with goat anti-mouse IgG or goat anti-rabbit secondary antibodies (Cell Signaling Technology, Danvers, MA, USA). The membranes were then washed with TBS-T buffer, visualized with Immobilon Western substrate (Millipore Corporation, Billerica, MA, USA), and detected with the ChemiDoc Touch Imaging System (Bio-Rad, Hercules, CA, USA). The band density was then normalized to the *β*-actin reference.

### 2.10. Statistical Analysis

Experiments were repeated at least three times with consistent results. Unless otherwise stated, data are expressed as the mean ± standard deviation of the mean. ANOVA was used to compare experimental values with control values. Comparisons between multiple groups were performed using Tukey's multiple comparison tests. The results were considered statistically significant at ^*∗∗∗*^*p* < 0.001.

## 3. Results

### 3.1. HPLC Analysis of ELT

**Figure 1:**
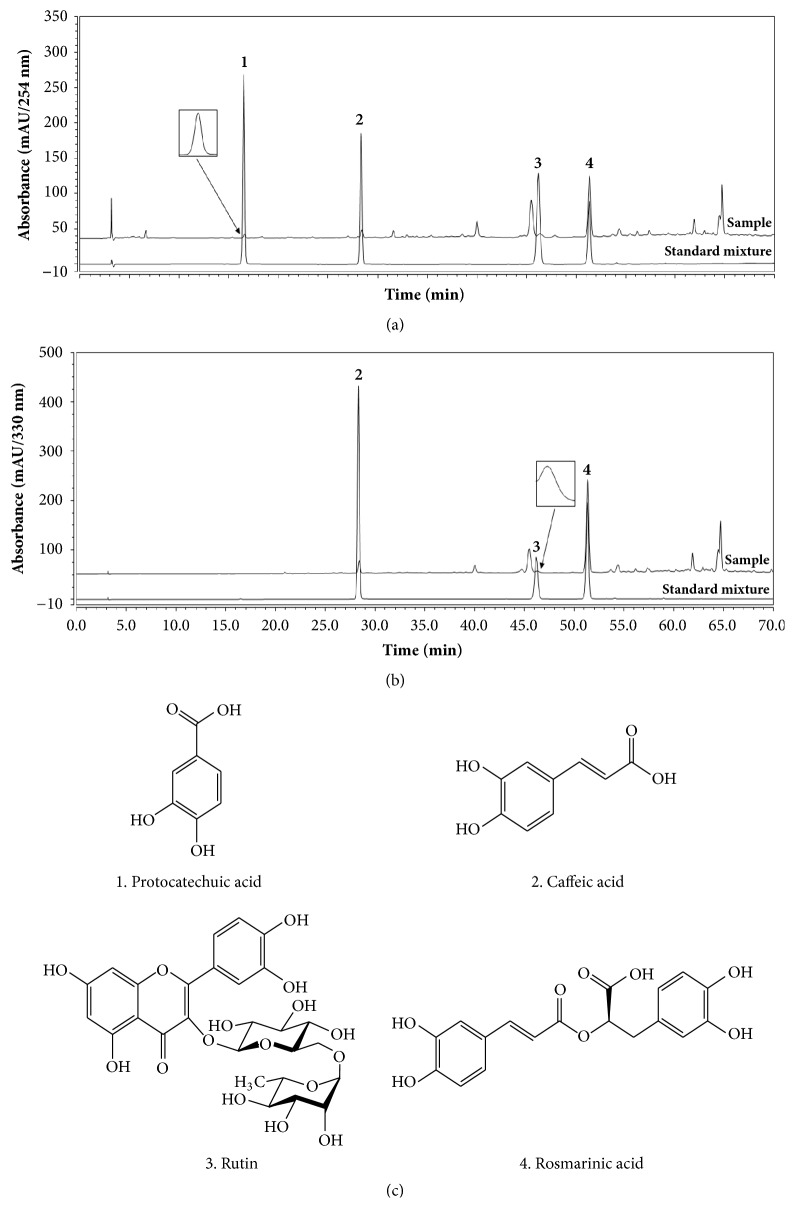
*High-performance liquid chromatography chromatogram and the chemical structure of 4 markers of ethanol extracts of Lycopus lucidus Turcz. ex Benth (ELT)*. (1) Protocatechuic acid, (2) caffeic acid, (3) rutin, and (4) rosmarinic acid. The markers were identified at wavelengths (a) 254 nm and (b) 330 nm using HPLC and diode array UV/VIS detector. (c) Chemical structure of four markers of ELT.

The four standard compounds of ELT were determined using HPLC analysis. According to the maximum absorption of the standard, our UV detector was set at 254 nm and 330 nm for the HPLC analysis of the four components: protocatechuic acid (1254nm), caffeic acid (2330nm), rutin (3330 nm), and rosmarinic acid (4330 nm). The identification of these four components in ELT was based on comparisons of their UV spectra, retention times (tR), and chromatographic patterns with those of the standards. All standards separated within 70 min and showed good selectivity without interference by other analyzed. As shown in Figure 1, the mixed standards (listed above) were detected by the chromatograph at retention times of (1) 16.52 min, (2) 28.33 min, (3) 46.20 min, and (4) 51.32 min. Under the same conditions, the retention times of the observed components in ELT were (1) 16.58 min, (2) 28.41 min, (3) 46.28, and (4) 51.35 min, respectively.

### 3.2. Effects of ELT on the Migration and Invasion of CT-26 Cells

**Figure 2:**
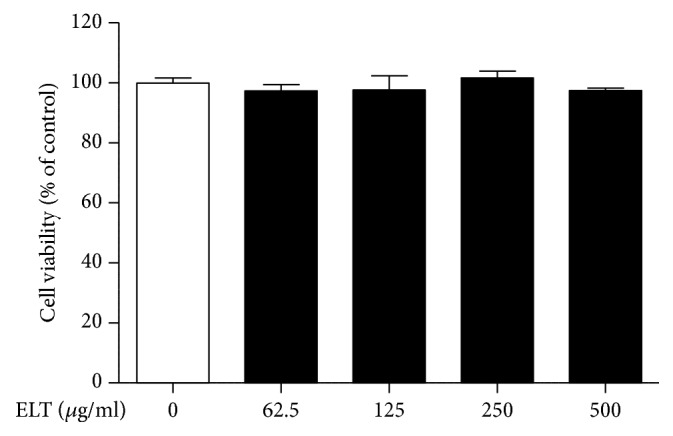
*Effect of ethanol extracts of Lycopus lucidus Turcz. ex Benth (ELT) on the viability of CT-26 cells.* Cells were treated with various concentrations (62.5–500 *μ*g/ml) of ELT for 24 h. Then cell viability was determined using a standard cell counting kit-8 assay. Cell viability is represented as the percent of relative absorbance relative to controls. Results are represented by the mean ± standard deviation of three independent experiments and compared using a t-test.

**Figure 3:**
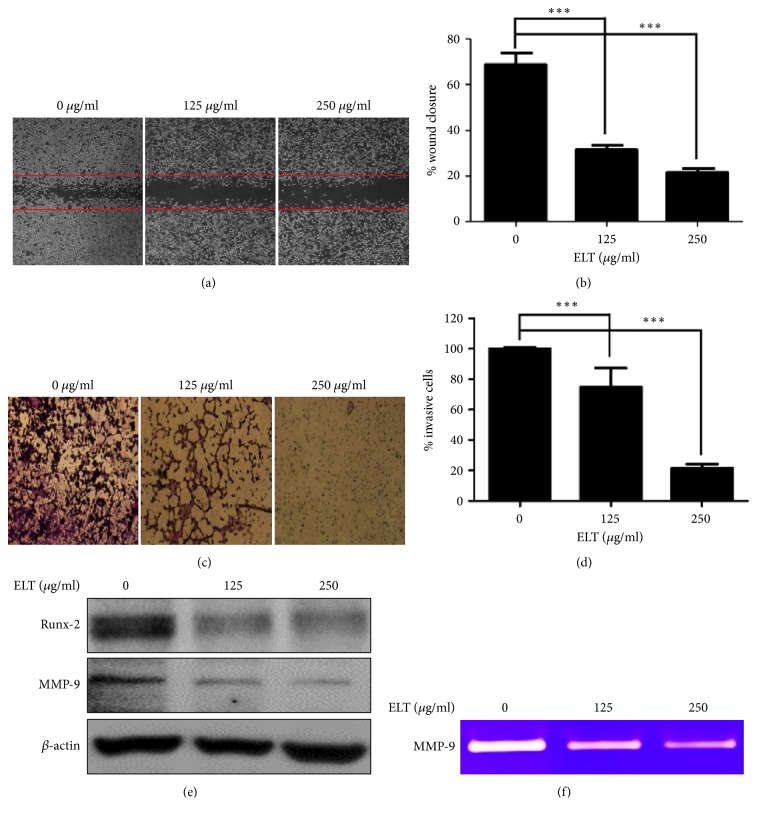
*Effects of ethanol extracts of Lycopus lucidus Turcz. ex Benth (ELT) on the migration and invasion of CT-26 cells.* (a) Effects of migration on ELT treated cells. (b) Densitometry of wound closure. Confluent cultures of CT-26 cells preincubated with 25 *μ*g/ml mitomycin C for 30 min and wounded with a micropipette tip, followed by treatment with ELT (125 and 250 *μ*g/ml) incubated at 37°C for 24 h. The cell migration index was calculated as the percent of wound closure. (c) Effects of invasion on ELT treated cells. (d) Densitometry of invasive cells. Invasion assay was performed using transwell, polycarbonate-membrane chambers with a 10-mm diameter, and an 8-*μ*m pore size after coating with 20 *μ*l of a 1:2 mixture of Matrigel:DMEM. Cells were suspended in serum-free DMEM and were then loaded onto the top chamber, after which ELT was added to specified concentrations (125 and 250 *μ*g/ml). Complete DMEM with 10% FBS was used in the lower chamber as a chemoattractant. After a 24 h incubation with ELT or without ELT, cells attached to the upper surface of the filters were removed by wiping with a cotton swab, and the filters were stained with 0.2% crystal violet/20% methanol (wt/vol) solution. Magnification was 200×. The relative degrees of migration and invasion were quantified using ImageJ. (e) Representative expressions for Runx-2 and MMP-9 proteins. (f) Representative zymography for the MMP-9 protein. CT-26 cells were treated with indicated concentrations of ELT for 24 h and then subjected to western blot analysis or gelatin zymography. Data are expressed as means ± standard deviation of three independent experiments. ^*∗∗∗*^*P* < 0.001 versus control group.

To determine the inhibitory effects of ELT, we first examined the viability of colon cancer CT-26 cells using CCK-8 assay. CT-26 cells were treated with ELT at our specified concentrations for 24 h. After 24 h, we confirmed that the effects of growth inhibition had not been altered (Figure 2). Because migration and invasion are important steps for cancer metastasis, we verified the antimetastatic effects of ELT using wound healing and transwell matrigel assays. As shown in Figure 3(a), ELT obviously inhibited wound closure in a dose-dependent manner, compared with the control. Wound closures were 31.37±1.20% at 125 *μ*g/ml and 21.48±0.85% at 250 *μ*g/ml. Moreover, ELT also significantly inhibited on cell invasion in a dose-dependent manner, consistent with our migration data (Figure 3(b)).

Several reports have demonstrated that the activation of Runx-2 is related to cancer progression and metastasis [6, 8]. In addition, the activation of Runx-2 targeted cells with migration and invasive metastatic-related genes, such as MMP-9 [19]. As shown in Figure 3(c), ELT significantly decreased the expression of Runx-2 and MMP-9 in a dose-dependent manner. Also, MMP-9 activity, confirmed using zymograpy, obviously decreased in a dose-dependent manner (Figure 3(d)).

### 3.3. Effects of Runx-2/MMP-9 Axis on the Migration and Invasion of CT-26 Cells

**Figure 4:**
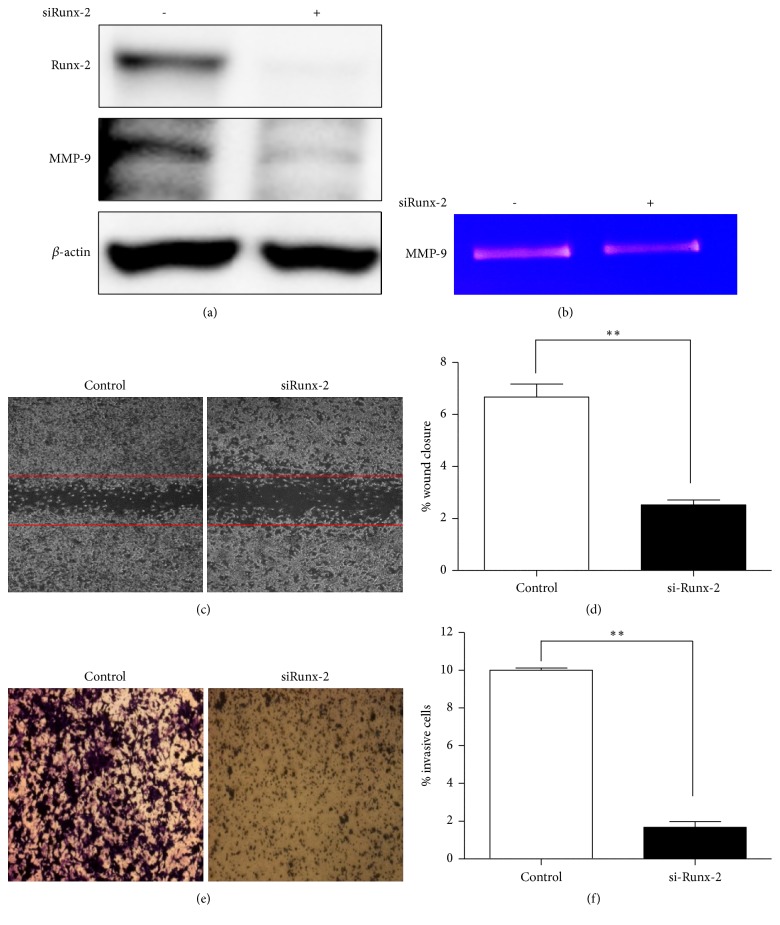
*Effects of runt-related transcription factor-2 (Runx-2)/matrix metalloproteinase- (MMP-) 9 on the migration and invasion of CT-26 cells*. (a) Effects of MMP-9 protein expressed on Runx-2 siRNA-transfected cells. (b) Effects of MMP-9 activities on Runx-2 siRNA-transfected cells. Cells were transfected for 24 h with specific Runx-2 siRNA (with fresh media without serum) and then subjected to western blot analysis or gelatin zymography. (c) Effects of migration on Runx-2 siRNA-transfected cells. (d) Densitometry of wound closure. (e) Effects of invasion on Runx-2 siRNA-transfected cells. (f) Densitometry of invasive cells. Cells were transfected for 24 h with specific Runx-2 siRNA (fresh media without serum) and then subjected to western blot analysis or gelatin zymography. Cells were transfected for 24 h with specific Runx-2 siRNA (fresh media without serum) and then analyzed for wound healing or an invasion assay. Magnification was 200×. The relative degrees of migration and invasion were quantified using ImageJ. Data are expressed as means ± standard deviation of three independent experiments. ^*∗∗∗*^*P* < 0.001 versus control group.

To better understand the mechanisms underlying Runx-2, we employed Runx-2 specific siRNA to suppress the expression of Runx-2 in CT-26 cells. As shown in Figure 4(a), MMP-9 expression suppressed Runx-2 by downregulation. Consistent with the western blot findings, MMP-9 activity also decreased (Figure 4(b)). Furthermore, the depletion of Runx-2 decreased wound closure. Specifically, wound closures were 66.71±2.88% and 25.30±1.06% relative to its insert vehicle and after si-Runx-2 treatment, respectively (Figure 4(c)). Also, the depletion of Runx-2 inhibited cell invasion in CT-26 cells (Figure 4(d)). These data indicate that Runx-2 modulates cell metastasis and invasion by increasing the level of MMP-9.

## 4. Discussion

Recent studies have indicated that ELT inhibits the vascular inflammation of human umbilical vein endothelial cells [15] and reduces mast cell-mediated allergies [16]. However, the antimetastatic effects of ELT-related to molecular mechanisms and relevant signal pathways in colon cancer are not yet fully understood. Importantly, this is the first report on ELT-inhibited Runx-2 protein expression. The aim of our study was to demonstrate the effects and underlying molecular mechanism(s) of ELT treatment on mouse colon carcinoma CT-26 cell lines.

Our results demonstrate that ELT does not inhibit cell viability in CT-26 cells but does significantly inhibit cell migration and invasion in a dose-dependent manner. To evaluate the possible mechanism(s) of the inhibitory activities of ELT on the invasion and migration of CT-26 cells, we determined the expressions of Runx-2 and MMP-9 after treatment with various concentrations of ELT. Runx-2 has been demonstrated to be an important transcription factor for osteoblast and skeletal morphogenesis and chondrocyte differentiation [7, 20]. Recently, extensive research has shown that Runx-2 is associated with metastasis of diverse types of cancers, such as breast and melanoma cancers, which are also potential targets for novel antimetastatic agents and diagnostic approaches for detecting cancer [21, 22]. In the present study, we demonstrated that ELT obviously decreases Runx-2 expression in a dose-dependent manner. It is well known that MMP-9 is related to the invasiveness and metastasis of a variety of cancer types [23, 24]. Furthermore, it is known that MMP-9 plays a critical role in epithelial mesenchymal transition [25, 26]. In our study, we confirmed that MMP-9 activation is inhibited by ELT treatment. Our results show that the antimetastatic effect of ELT is associated with inhibition of the degradation processes of metastasis. Therefore, it is possible that ELT may regulate bone homeostasis through the regulation of Runx2 and MMP-9 expression.

Furthermore, we used Runx-2 specific siRNA to further determine the relationship between Runx-2 and MMP-9 in the migration and invasion of CT-26 cells. Several studies have indicated that Runx-2 regulates MMP-9 expression in metastatic cancer cells [8]. Our findings demonstrate that the downregulation of Runx-2 expression inhibits MMP-9 expression and activities. In addition, the depletion of Runx-2 decreases its migration and invasion in CT-26 cells. These findings are consistent with results showing that ELT decreases metastasis by inhibiting Runx-2 and that these results agree with MMP-9 inhibition. Further investigations are required to determine the underlying molecular mechanisms of ELT in relation to Runx-2 and MMP-9 inhibition.

Our results suggest that ELT inhibits cell metastasis and invasion of colon cancer CT-26 cells and may provide an alternative strategy for the treatment of colon cancer. Therefore, ELT may be a novel anticancer agent that can confer protection against cell metastasis.

## Data Availability

All data used to support the findings of this study are included in the article.

## Abbreviations

LT: Lycopus lucidus Turcz
ELT: Ethanol extracts of Lycopus lucidus Turcz
Runx2: Runt-related transcription factor 2
MMP-9: Matrix metallo proteinase 9
HPLC: High-performance liquid chromatography
DMEM: Dulbecco's modified Eagle's medium
FBS: Fetal bovine serum
CCK-8: Cell counting kit-8 assay
SDS: Sodium dodecyl sulfate
SDS-PAGE: SDS-polyacrylamide gel.
